# Life Prediction Method of Dissimilar Lightweight Materials Welded Joints with Precrack under Coupled Impact-Fatigue Loading

**DOI:** 10.3390/ma15145077

**Published:** 2022-07-21

**Authors:** Zhengshun Ni, Tao Xiong, Jie Lei, Liuping Wang, Tong Gao, Jianwu Yu, Chengji Mi

**Affiliations:** 1 Department of Mechanical Engineering, Hunan University of Technology, Zhuzhou 412007, China; happy2022hut@126.com (Z.N.); xiongt@126.com (T.X.); leijie2022@126.com (J.L.); wanglp@126.com (L.W.); gaotong@126.com (T.G.); 2 Department of Mechanical and Vehicle Engineering, Hunan University, Changsha 410082, China; mcj20112011@hnu.edu.cn

**Keywords:** welded joints, continuum damage mechanics, fatigue life, impact damage

## Abstract

This paper aims to explore the fatigue life estimation approach of welded joints with precrack under coupled impact and fatigue loading, and the base metal is dissimilar 5083H111 and 5754 aluminum alloy. Impact tests are first carried out on the dissimilar lightweight materials welded joint with precrack located in the middle of the specimen, and a stress and strain field is obtained to determine the fatigue damage model parameters by using finite element dynamic analysis to simulate the impact process. Based, on the S-N curve of welded joints, the predicted life expectancy is found to be inconsistent with the experimental results. According to the continuum damage mechanics, the lifetime assessment model is presented to calculate both impact and fatigue damage. The estimated results agree well with the experimental ones.

## 1. Introduction

Lightweight materials such as aluminum and magnesium alloys are widely used in automotive parts, marine connection bodies, rail transportation passing structures, and other engineering components due to its series of excellent characteristics such as low density, high stiffness, and good corrosion resistance [1]. Lightweight metals of engineering practical structures are usually connected by welding joints. However, the welding process generates residual stresses and defects such as porosity and incomplete penetration, which are prone to produce macroscopic crack under fatigue or impact loading [2,3]. Therefore, it is important to reveal the fatigue failure mechanism of dissimilar light metal welded joints including initial defects under complex loadings [4,5].

In recent years, in order to predict the remaining fatigue life of welded joints with precrack, many lifetime assessment methods have been developed by researchers. For example, the critical plane method was extended from two-dimensional to three-dimensional by Peng Luo [6]. This method predicted the fatigue life by defining the critical plane of welded joints with precrack as the maximum shear stress plane, while the Susmel parameter was combined with the semi-empirical formula [7]. However, the parameters in this method lacked a clear physical meaning, and the stress at the notch made it difficult to exactly determine in complex loading situations. Some people considered that any defect was a “weak point” and could directly affect the strength of the structure [8,9,10]. Then, the parameters that could define local damage were proposed, and it was assumed that the lifetime of defective specimen was equal to the smooth specimen if they had the same historical stress field strength. However, this method depended too much on a large amount of test data to determine the size of the damage area. A fatigue life prediction method based on the local mechanical responses was proposed by P. A. Fomichev [11,12], which can predict the fatigue life of material based on the local stress and strain and the fatigue properties of the material, but it was more applicable to assess the lifetime with the simple geometric configuration [13].

In this paper, welded joints with the parental material 5083H111 and 5754 aluminum alloy, including U-shaped precrack in the middle of specimen, were designed, and fatigue tests were conducted on the specimens after impact loading. Then, the finite element dynamic analysis was utilized to simulate the impact process for determining the stress and strain field of welded joints. The predicted fatigue life of this dissimilar welded joints were performed by the S-N approach and the suggested model was based on the resulting continuum damage mechanics.

## 2. Experimental Research

### 2.1. Mechanical Properties

**Figure 1:**
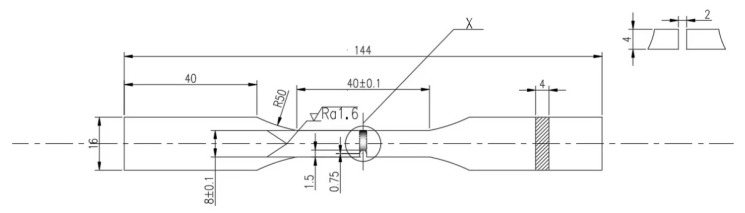
Dimension of dissimilar lightweight materials welded joint with precrack (unit in mm).

In this paper, material 5083H111 and 5754 aluminum alloys are considered as the base metal for the welded joints to study the damage mechanism under impact and fatigue loadings, due to their low modulus of elasticity and good ability to absorb an impact-effect. Their chemical composition and mechanical properties are shown in Table 1 and Table 2 [14,15], respectively. The AC argon arc welding are used to produce the weld. The total length of the specimen is 144 mm and the thickness is 4 mm. The weld area is located in the middle of the specimen. According to the Charpy pendulum impact test standard [16], the U-shaped precrack with depth of 1.5 mm, width of 1.5 mm and top radius of 0.75 mm is designed in the center of the specimen, as shown in Figure 1.

### 2.2. Impact Test

For the bearing welded structure, it may have some defects such as micro-crack and has to suffer the impact loading during in-service [17]. The impact test is designed to simulate the damage caused by the falling object from a direction perpendicular to the specimen, while the pre-crack in the welded joint is back to the impacted surface. In this impact test, the specimen is considered as an elastic body, and the rigid ball with weight of 350 g will start the free-falling movement at a height of 800 mm. In order to protect the tester, a hollow tube is fixed above the test piece, and the steel ball with the radius of 35 mm falls down along its axis. The test bench is designed and made by the author. The ends of the specimen are fixed, and the middle part is impacted by the rigid body ball. Considering that the stiffness of the impacting foreign object is much higher than structural material stiffness, it is reasonable to regard the foreign object as a rigid body and the structure as an elastoplastic body [18]. The impact test steps are as follows: fixing the above specimen at a certain place; using a rigid ball free fall onto the specimen; and causing the specimen to deform on impact. Although the impact test is simple, it can characterize the state of the component well after it has been damaged.

**Figure 2:**
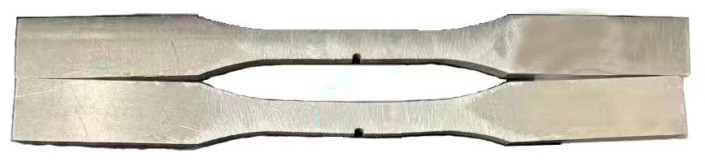
Comparison of deformed specimens of dissimilar lightweight materials welded joints with and without impact.

**Figure 3:**
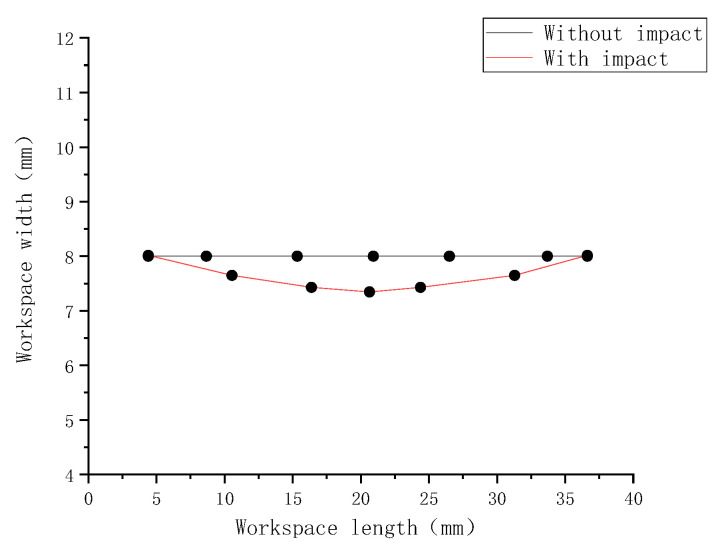
Comparison of deformation results of specimens of dissimilar lightweight materials welded joints along the width direction.

The specimen has bending deformation after impact, shown in Figure 2. This deformation is irreversible, and results in impact damage. The specimens with and without impact are compared in Figure 2. In order to facilitate the observation of the changes in the specimen, the point at the leftmost bottom of the specimen is considered as the origin to establish the coordinate axis, and then the planar deformation results on the impacted surface of the workspace for the two specimens are displayed in Figure 3. It can be clearly seen that the maximum deformation on the impacted surface becomes 0.7 mm. This indicates that the impact damage produces plastic deformation.

### 2.3. Mechanical Properties of Specimens after Impact Test

**Figure 4:**
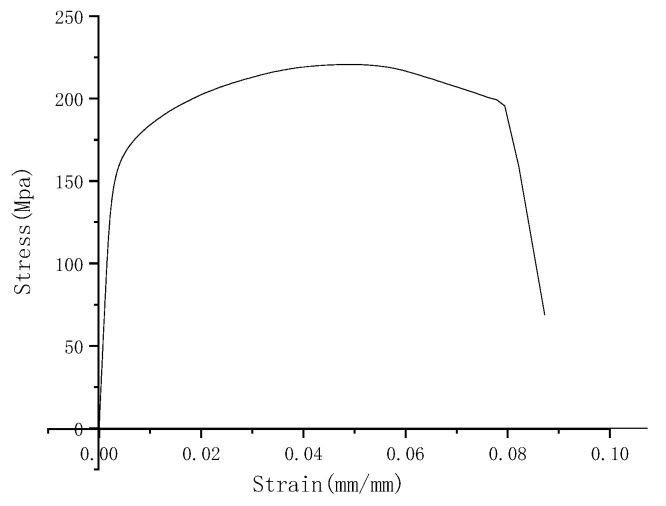
Stress-strain curve of dissimilar lightweight materials welded joints with precrack after impact loading.

The plastic damage caused by the impact loading has a great impact on the mechanical properties of the material, and the mechanical parameters of the welded joints are determined through the monotonic tensile test [19]. PLD-100 microcomputer-controlled electro-hydraulic servo universal testing machine is used for the tensile test. The monotonic tensile test is performed at a constant force loading rate of 200 N/s, while the testing environment is at room temperature, according to the Chinese testing standard of GB/T228.1-2010 [20]. The test is stopped until the maximum loading force is half-reduced. The stress-strain curve of the specimen is obtained, as shown in Figure 4, and the mechanical properties of the welded joint are listed in Table 3. Compared with results without impact obtained from Table 2, the elastic modulus of the welded joints after impact loading is a little less than the one without impact. The impact loading may result in the hardening of the welded joints, so the yield strength and tensile strength of the impacted specimen are a little higher as well.

### 2.4. Fatigue Test

Before the fatigue test, the specimen of welded joints with impact damage is carefully polished with sandpaper to improve the surface quality. The fatigue tests are under the stress control, and the stress level distribution is considered as 100 MPa, 140 MPa, 180 MPa, respectively. According to the standard GB/T15248-2008, the stress ratio R is 0.1, and the loading frequency is 1 Hz. All tests run at room temperature. The number of fatigue life cycles for the specimen at different stress levels is listed in Table 4.

**Figure 5:**
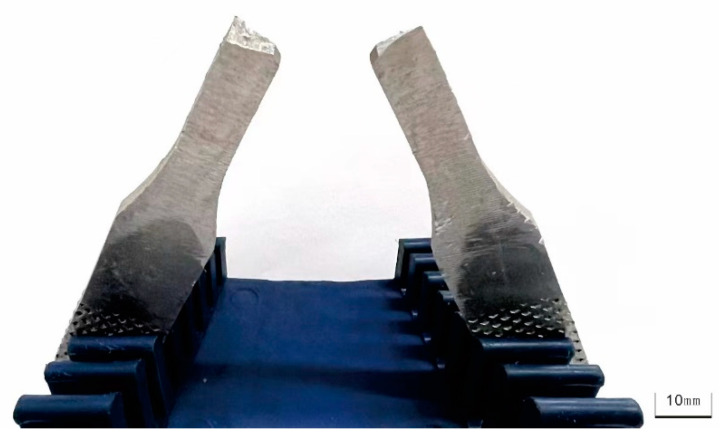
Fatigue fracture morphology of specimen No. 1 of dissimilar lightweight materials welded joints.

Figure 5 shows the fatigue fractographs of the No. 1 specimen of dissimilar lightweight materials after cyclic loading, and it can be seen that the fatigue cracks sprout from the impacted surface and spread to the root of the precrack. These results show that the impact damage has a great influence on the cumulative fatigue damage, and even produce more contributions to the macro-crack than the precrack.

## 3. Finite Element Simulation

### 3.1. Finite Element Modeling

**Figure 6:**
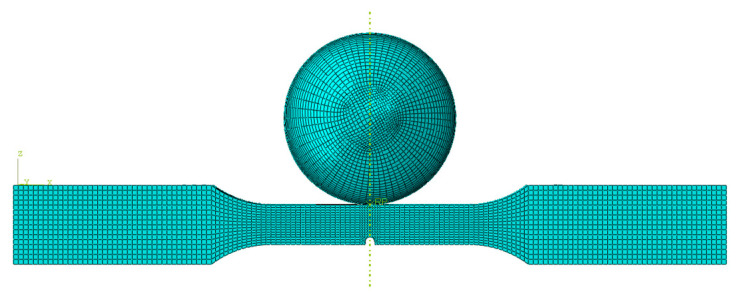
Finite element model of dissimilar lightweight materials welded joint and rigid ball for the impact process.

In order to obtain the stress and strain field through a more detailed dynamic analysis of the impact test, the finite element simulation is considered to analyze the impact process. In this paper, software ABAQUS is used to establish the finite element model of the specimen as an elasto-plastic body, and the impact sphere is set as a discrete rigid body. The material parameters of finite element model are given from Table 2 and Table 3. In order to ensure the computational accuracy and improve computational efficiency, the number of elements near the impact region is locally increased, and the model has a total of 19,168 elements. The element type is eight-node linear hexahedral cell (C3D8R), as shown in Figure 6.

### 3.2. Impact Numerical Analysis

**Figure 7:**
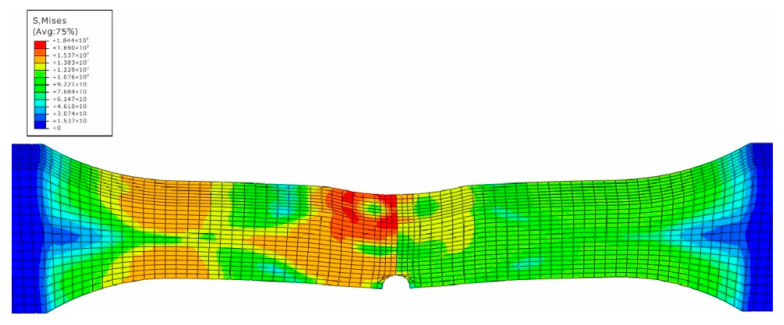
Stress field of impacted surface of dissimilar lightweight materials welded joint.

**Figure 8:**
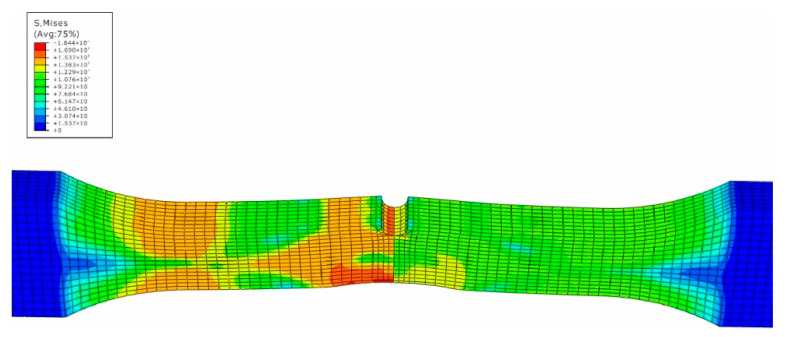
Stress field of back surface with precrack of dissimilar lightweight materials welded joint.

**Figure 9:**
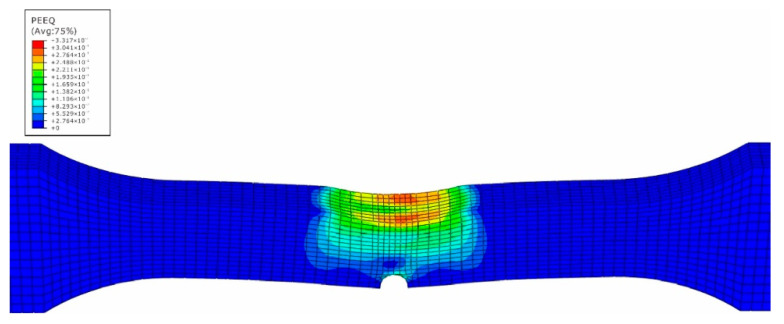
Strain field of impacted surface of dissimilar lightweight materials welded joint.

**Figure 10:**
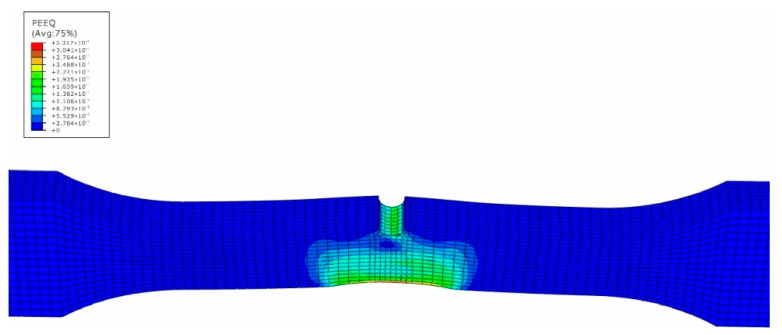
Strain field of back surface with precrack of dissimilar lightweight materials welded joint.

The impact numerical analysis is performed by a velocity-controlled manner. The specimen is completely fixed at two ends. In order to save the simulation time, the impacting rigid ball is given an initial velocity instead of simulating the whole free fall process. The stress and strain field of the specimen is shown in Figure 7, Figure 8, Figure 9 and Figure 10.

In Figure 7, the maximum stress caused by the impact loading is located at the side of material 5083H111 along the middle line of the specimen, while the high stress region also attends 10 mm away from the center line within the side of the base metal 5083H111. On the contrary, the maximum equivalent strain appears at the intersection region of the edge and the middle line within the side of material 5754, and its value is 0.33 mm/mm. From Figure 3, one can see htat the tested maximum strain is 0.30 mm/mm, which means the simulated results match well with the experimental data. The precrack tip has high stress and strain as well, but is not the biggest. This means that the impacted surface has impacting damage, as well as the precrack region. This is caused by the difference of the elastic-plastic properties between the two materials to produce the complex and nonlinear stress and stain field [21]. Based on the simulated results, the impacted and the pre-cracked area are prone to have fatigue failure due to the high stress and strain caused by the impact loading.

**Figure 11:**
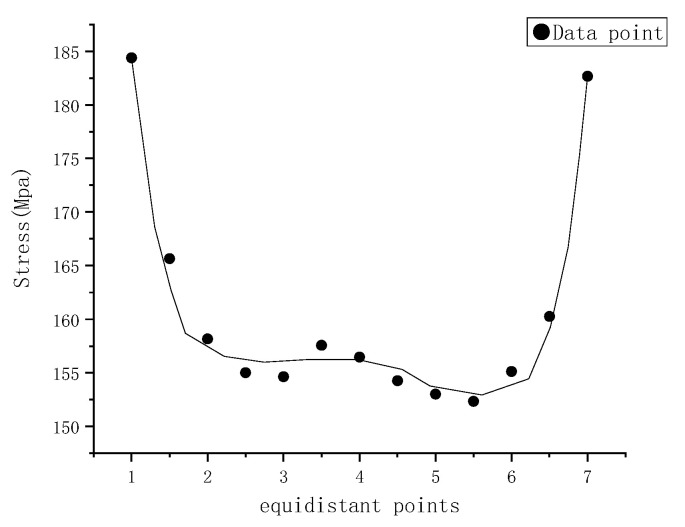
Stress distribution of data points of dissimilar lightweight materials welded joint along thickness direction.

**Figure 12:**
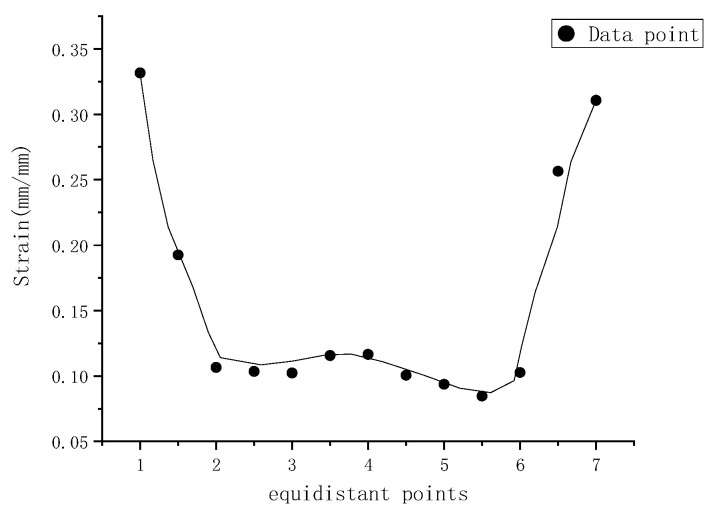
Strain distribution of data points of dissimilar lightweight materials welded joint along thickness direction.

In order to qualify the stress and strain caused by the impact loading for the whole welded part through-thickness, the equidistant thirteen points from the top impacted surface to the bottom precracked surface are selected to obtain the mechanical responses from the simulated results, as shown in Figure 11 and Figure 12. The beginning point is the maximum stress location on the impacted surface, while the ending one is the highest stress point on the precracked surface. It could be seen from Figure 11 that the largest stress is around 185 MPa, and the stress level between the two surfaces is around 160 MPa. The similar phenomenon is found in the strain distribution from Figure 12. By the way, the strain value is obtained from the spot the same as the stress point. Based on the simulated results, the high stress and strain region and precracked location may both be a potential fatigue failure area [22].

## 4. Fatigue Life Prediction Method

### 4.1. S-N Curve Life Assessment

**Figure 13:**
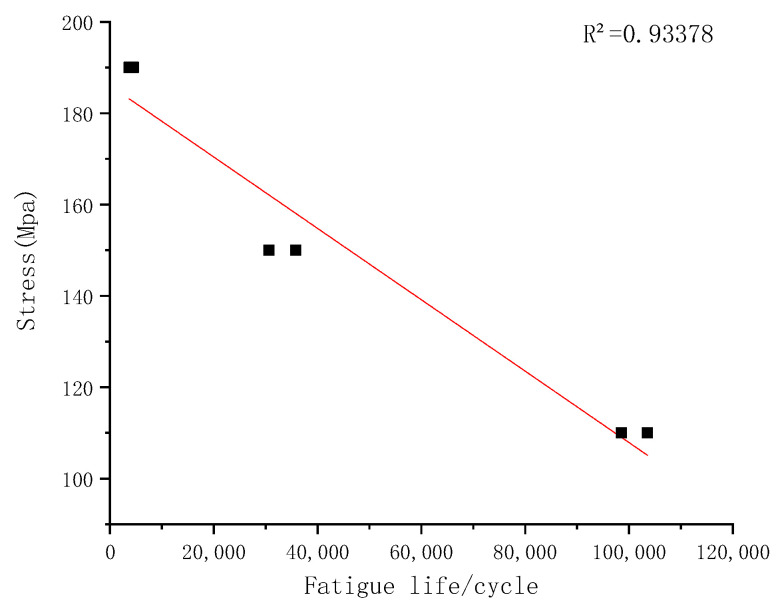
S-N Curve of welded joints of dissimilar lightweight materials welded joints.

**Figure 14:**
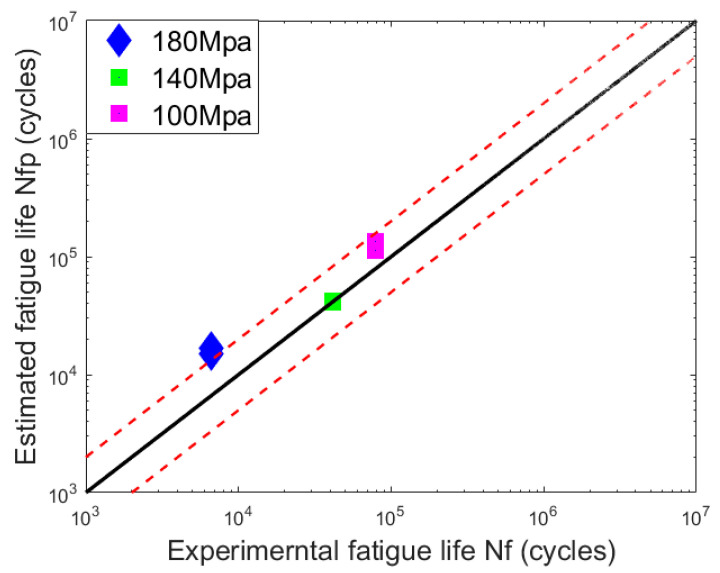
Fatigue life prediction of dissimilar lightweight materials weld joints from S-N curve method.

The S-N curve is a life prediction method developed on the basis of stress and has widely contributed to the quantitative description of metal fatigue. Based on the number of fatigue life cycle for the welded joints and the relative stress level, the S-N curve of the welded joints is determined, as shown in Figure 13. The predicted fatigue life of welded joints based on the S-N curve is compared with the experimental data, as shown in Figure 14, and the red dashed line is the two-time life scattering band. From Figure 14, one can see that the estimated lifetime for two low stress levels is within the scattering band, but the assessed result for the stress level 180 MPa is beyond the scattering band. Therefore, another approach is needed to predict the fatigue life of the welded joints with pre-crack and impact damage.

### 4.2. Life Prediction Based on Continuum Damage Mechanics

#### 4.2.1. Plastic Damage Model

In order to exactly estimate the lifetime of welded joints, the continuum damage mechanics is used to quantify the coupled impact and fatigue damage. The basic concept of continuum damage mechanics was proposed by Lemaitre and Chaboche in 1994. Based on the framework of continuum damage mechanics, the plastic deformation and failure of the isotropic material can be expressed by the damage variable D (i.e., [23]):

(1) $$D=\frac{E-E_{D}}{E}$$

E and $E_{D}$ denote the Young’s modulus and the effective Young’s modulus of the material after damage, respectively, and the values of $E_{D}$ range from 0 to E, and the value of E range from 0 to 1. The damage variable D can include plastic damage, creep damage, fatigue damage, etc. The impact loading described in this paper produces a sufficient amount of plastic deformation, which can be categorized as plastic damage. A Lemaitre plastic damage model based on damage variables, plastic strain rate, and stress state was proposed in a subsequent damage study [23] as follows:

(2) $$\dot{D}={\left[\frac{\sigma_{eq}^{2}R_{v}}{2ES{\left(1-D\right)}^{2}}\right]}^{S_{0}}\dot{P}$$

$\sigma_{eq}$ is the equivalent stress, $\dot{P}$ is the cumulative plastic strain rate, and S and $S_{0}$ are material parameters. $R_{V}$ is the triaxial stress function, and is described as follows [23]:

(3) $$R_{V}=\frac{2}{3}\left(1+V\right)+3\left(1-2V\right){\left(\frac{\sigma_{H}}{\sigma_{eq}}\right)}^{2}$$

where V is Poisson’s ratio, $\sigma_{H}$ is hydrostatic stress, and $R_{V}$ is equal to 1 in the uniaxial stress state.

Integrating Equation (2) with the plastic strain process, the initial damage of the welded joint caused by the external forces can be obtained [23]:

(4) $$D_{0}={\left[\frac{\sigma_{eq\max}^{2}R_{v}}{2ES_{0}}\right]}^{S_{0}}\Delta P$$

where ΔP is the total value of the accumulated plastic strain during the whole impact process.

#### 4.2.2. Fatigue Damage Model

In 1988, a cumulative fatigue damage evolution equation for the uniaxial stress case was proposed by Chaboche, J. and Lesen, P. as follows [24]:

(5) $$\dot{D}=\frac{dD}{dN}={\left[1-{\left(1-D\right)}^{\beta +1}\right]}^{\alpha }{\left[\frac{\sigma_{a}}{M\left(\sigma_{m}\right)\left(1-D\right)}\right]}^{\beta }$$

where N is the number of cycles, $\sigma_{a}$ is the maximum applied stress for cyclic loading, $\sigma_{m}$ is the average stress, β is the material constant, α and $M\left(\sigma_{m}\right)$ can be defined as [24]:

(6) $$\alpha =1-a\frac{\sigma_{a}-\sigma_{0}}{\sigma_{b}-\sigma_{a}}$$

(7) $$M\left(\sigma_{m}\right)=M_{0}\left(1-b\sigma_{m}\right)$$

where, $\sigma_{b}$ is the ultimate tensile stress, $\sigma_{0}$ is the fatigue limit of the material, a, $M_{0}$, b are material constants. 〈σ〉 is the Macaulay brackets. 〈σ〉 can be defined as: if 〈σ〉 is greater than 0, then 〈σ〉 = σ; if 〈σ〉 is less than or equal to 0, then 〈σ〉 = 0. then 〈σ〉 = 0.

The uniaxial fatigue life $N_{f}$ (corresponding to *D* = 1) can be obtained by integrating from *D* = $D_{0}$ to *D* = 1 for the damaged material as follows:

(8) $$N_{f}=\frac{1}{\left(1-\alpha \right)\left(1+\beta \right)}{\left[\frac{M\left(\sigma_{m}\right)}{\sigma_{a}}\right]}^{\beta }\left\{1-{\left[1-{\left(1-D_{0}\right)}^{1+\beta }\right]}^{1-\alpha }\right\}$$

### 4.3. Fatigue Life Prediction Based on the Suggested Model

Before estimating the life expectancy of welded joints with the coupled impact and fatigue damage, the parameters in Equation (8) have to be determined firstly. It includes that the elastic-plastic parameters of the welded joints after impact damage, which can be obtained from the monotonic tensile test. The impact damage model material performance parameters, *S* and $S_{0}$ can be defined by the experimental data of low cycle fatigue tests on nonimpacted specimens. The fatigue performance parameters in the fatigue damage model, α, β, $M_{0}$, and *b*, can be determined by the experimental results of the high cycle fatigue tests on nonimpacted specimens. The parameters are listed in Table 5.

**Figure 15:**
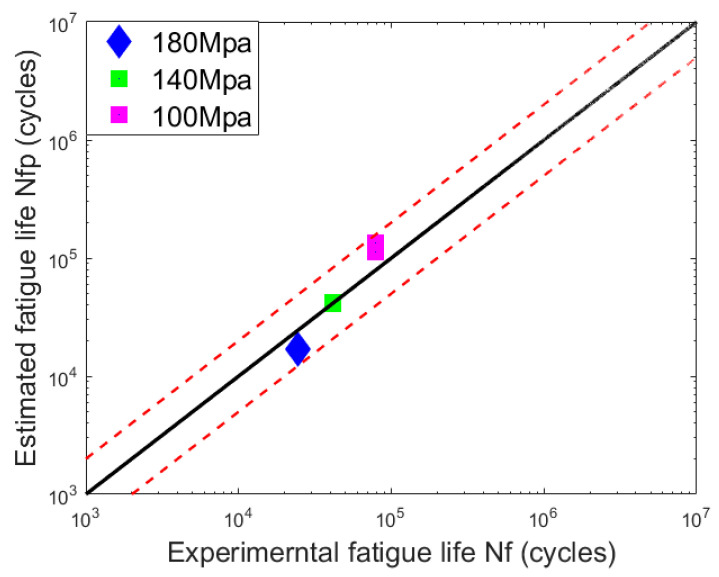
Fatigue life prediction of dissimilar lightweight materials welded joints from continuum damage mechanics method.

Then, the fatigue life of welded joints based on this suggested model can be conducted on the specimens, as shown in Figure 15. All of the predicted data stays within the two-time life scattering band, and the result for the stress level 140 MPa is very close to the middle line, which stands for that the predicted lifetime is equal to the tested data.

According to the estimated results, the lifetime prediction model based on the continuous damage mechanics is significantly better than the results obtained from the S-N curve method. This means that the suggested model can give more satisfactory results when predicting the fatigue life of the welded joints with precrack for the coupled impact and fatigue damage.

## 5. Conclusions

In this paper, the impact tests are conducted on the welded joints with precrack, and the base material is 5083H111 and 5754 aluminum alloy. The plastic damage is evaluated by finite element simulation of the impact process. The high stress area is 10 mm away from the middle line at the side of material 5083H111, while the high strain region appears at the intersection part of the edge and the middle line at the side of material 5754. The stress and strain distribution along the thickness is also determined. The deformation from the impact data is in good agreement with the simulated ones, which provides a good reference for the subsequent calculation of the life expectancy prediction.

The S-N fatigue life prediction model and the suggested model based on continuum damage mechanics are both used to predict the remaining lifetime of the welded joints with precrack after impact. The suggested prediction model is more accurate, and all predicted data stay within the two times scattering band.

## Figures and Tables

**Table 1:** Main chemical composition of two aluminum alloys (%).

	Mg	Mn	Cr	Si	Cu	Ti	Fe	Zn
5083H111	4.50	0.60	0.12	0.20	0.15	0.02	0.30	0.02
5754	3.40	0.50	0.01	0.10	0.04	0.04	0.30	-

**Table 2:** Mechanical properties of two aluminum alloys and its welded joints.

	Elastic Modulus (GPa)	Yield Strength (MPa)	Tensile Strength (MPa)	Poisson’s Ratio
5083H111	69.50	139.00	300.00	0.33
5754	61.00	117.00	195.00	0.31
Welded joints	72.30	136.00	206.90	0.31

**Table 3:** Mechanical properties of specimens after impact loading.

Material Properties	Elastic Modulus (GPa)	Yield Strength (MPa)	Tensile Strength (MPa)	Poisson’s Ratio
	64.90	164.00	220.60	0.33

**Table 4:** Number of fatigue life cycle for the welded joints.

Nominal Stress/MPa	Specimen Number	Fatigue Life of Welded Joints/Cycle	Average Life Span/Cycle
100	1	134,420	123,086
	2	111,752	
140	3	40,882	41,642
	4	42,402	
180	5	15,114	13,906
	6	12,698	

**Table 5:** Material parameters.

*S*	$S_{0}$	α	β	$M_{0}$	*b*
1.1	3.8	0.969	1.6	75,000	0.0011

## Data Availability

Not applicable.
